# Editorial: Immunity in Marine Invertebrates: Integrating Transcriptomics to Proteomics and Metabolomics

**DOI:** 10.3389/fimmu.2021.755839

**Published:** 2021-09-01

**Authors:** Antonio Figueras, Rita Marino, Daniela Melillo, Annalisa Pinsino

**Affiliations:** ^1^Institute of Marine Research (IIM), National Research Council (CSIC), Vigo, Spain; ^2^Biology and Evolution of Marine Organisms (BEOM), Stazione Zoologica Anton Dohrn (SZN), Naples, Italy; ^3^Institute of Biochemistry and Cell Biology (IBBC), National Research Council (CNR), Naples, Italy; ^4^Institute for Biomedical Research and Innovation (IRIB), National Research Council (CNR), Palermo, Italy

**Keywords:** marine invertebrates, immune response, transcriptomics, proteomics, metabolomics

Marine invertebrates defense strategies are heterogeneous in terms of effector mechanisms and are strictly correlated to the surrounding environment challenges. Studying the cellular and molecular mechanisms involved in the immune performance of marine invertebrates is crucial to figure out the way they deal with biotic and abiotic stressors and helps to better understand the whole animal physiology in different environmental settings. Marine invertebrates lack adaptive immune responses, yet some of them show specific genetic mechanisms known to increase the diversity and specificity of the innate immune responses (1, 2), and mainly rely on innate immunity. The marine invertebrate immune system mainly encompasses 1) physical and chemical barriers that prevent the entry of pathogens into the body and 2) specialized cells and humoral factors that avoid the spread of infections, as well as mount a repertoire of actions that reduce or remove the potential damage. Several marine invertebrates (eg., sea urchin, oyster, mussel) present highly complex innate immune systems with discriminatory properties and responses to both pathogens and environmental stressors, including bacteria, viruses, and pollutants. More importantly, among marine invertebrates there are sessile species (eg. hydroids, sponges, sea squirts) which can escape both biotic and abiotic stressors only by modulating their immune system, ensuring survival.

The seven scientific papers that contributed to the Research Topic “Immunity in Marine Invertebrates: Integrating Transcriptomics to Proteomics and Metabolomics” provided an overview of the wide-ranging approaches currently available to highlight mechanisms required for the function and regulation of the marine invertebrate immune system, including cell biology, biochemistry, and physiology advanced techniques (i.e., proteomics, metabolomics, genomics, transcriptomics).

Omics represent a powerful tool in understanding the impact of environmental challenges on the marine invertebrate immune system as the interaction between host, environment, and pathogens require a combination of both functional assays and cutting-edge technology able to detect any change in immune homeostasis induced by environmental contaminants (**Figure 1**). Among marine invertebrates, bivalves are extensively recognized as one of the main suitable “sentinel” organisms for risk assessment in environmental toxicology (Balbi et al.) and resilience studies to biotic and abiotic stress (Moreira et al.). The application of transcriptomics in (eco)toxicology has been successfully applied to bivalve immunity to measure, evaluate, and predict exposure-related health risks and individual susceptibilities (exposome), as reviewed by Balbi et al., as well as to investigate the molecular basis of the innate immune responses against pathogen-associated molecular patterns (e.g., poly I:C, β-glucans, LPS) (Moreira et al.). After a bath infection, mussels are able to remove the bacteria from their bodies and the water, and gills play a central role in removing and clearing potential pathogens, activating the effector agents of the immune response to overcome bacterial infection (Saco et al.).

**Figure 1:**
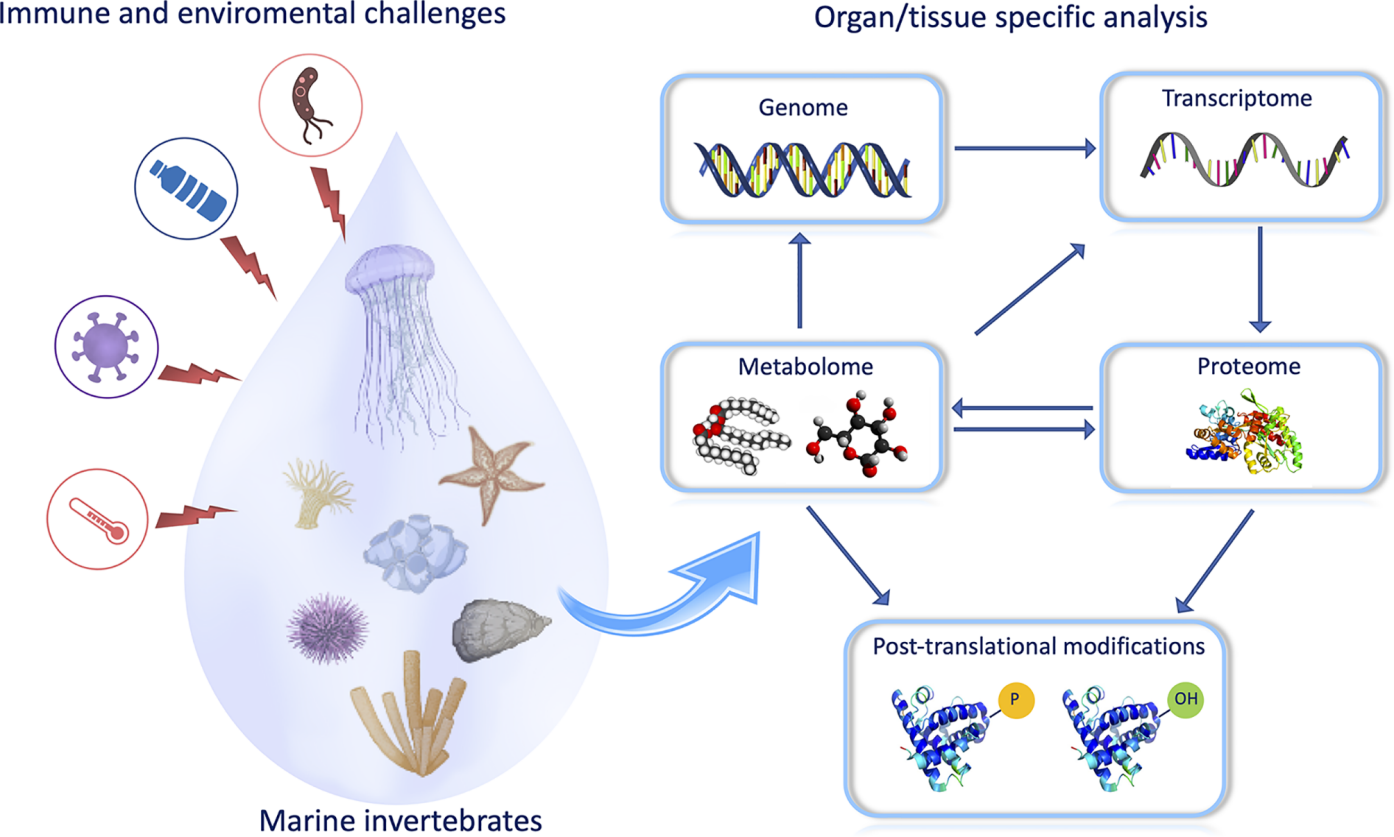
The study of biological systems/processes has focused on one aspect of the system, such as genomics, transcriptomics, proteomics, and metabolomics. A synthesis of all information from these multi-omics approaches is required to provide a full understanding of an immune response. For example, the correlation of appearance and/or accumulation of some metabolites with protein modifications could allow to build up the pathways engaged by animals to respond to a specific challenge (T. Heinzl provided the scheme of the figure).

Shotgun proteomics was applied to unravel the molecular mechanisms underlying the differences in susceptibility to OsHV-1 of two oyster families (Lepretre et al.). Interestingly, further to validate the transcriptomic data indicating an antiviral response mediated by interferon-like and RNAi pathways in more resistant oysters, this approach brought out an accumulation of lysosomal proteins in susceptible oysters’ family, as the OsHV-1 blocked the fusion of the autophagosome with the lysosome to escape the immune response. Thus, the comparative analysis of protein response, combined with the immune phenotype, allowed us to hypothesize that the cell function is impaired in a more sensitive families.

The study from Frizzo et al. encourages the use of NMR-based metabolomics as an integrative tool to investigate physiological adjustments occurring in stressed and immune-stimulated animals. Moreover, the authors point out that the experimental design is the critical issue of all studies carried out on animals as a whole.

An interesting approach to focus on the interplay between stem cells and the immune system for understanding the physiology and ecology of the organisms and contributing to (eco)toxicology and medicine aspects in a more integrative and transformative way, was discussed by Ballarin et al.. Stem cells originate immune cells through hematopoiesis and both cells contribute to maintaining homeostasis, therefore, the idea to relate stem and immune cells starting with invertebrate models (e.g., mollusks, Crustacean, echinoderms, ascidians) open a new framework to address immunological mechanisms in an unusual but promising view.

To decipher the relationships between microbes and host physiology, Liberti et al. presented the invertebrate chordate *Ciona robusta* as a recent host-microbiome model system. The authors reviewed recent literature on the characterization of the C*iona* gut environment, focusing on the structure and organization of the mucus layers and the immune molecules produced by the epithelium. They reported the microbiota composition and the functional characterization of some interactions between different elements within the gut environment. To further elucidate the mucus-microbiota and immune-microbiota interactions they proposed the use of integrating multi-omics technologies. These combined approaches would clarify the basic mechanisms of host-microbial interactions that shape animal physiology and, ultimately, health and disease.

## Author Contributions

All authors have contributed equally to this work. All authors contributed to the article and approved the submitted version.

## Conflict of Interest

The authors declare that the research was conducted in the absence of any commercial or financial relationships that could be construed as a potential conflict of interest.

## Publisher’s Note

All claims expressed in this article are solely those of the authors and do not necessarily represent those of their affiliated organizations, or those of the publisher, the editors and the reviewers. Any product that may be evaluated in this article, or claim that may be made by its manufacturer, is not guaranteed or endorsed by the publisher.
